# An innovative dual recognition aptasensor for specific detection of *Staphylococcus aureus* based on Au/Fe_3_O_4_ binary hybrid

**DOI:** 10.1038/s41598-022-15637-1

**Published:** 2022-07-22

**Authors:** Mohamed M. El-Wekil, Hamada Mohamed Halby, Mahmoud Darweesh, Mohamed E. Ali, Ramadan Ali

**Affiliations:** 1 Department of Pharmaceutical Analytical Chemistry, Faculty of Pharmacy, Assiut University, Assiut, 71524 Egypt; 2 Department of Microbiology and Immunology, Faculty of Pharmacy, Al-Azhar University, Assiut Branch, Assiut, 71524 Egypt; 3 Department of Medical Biochemistry and Microbiology, Uppsala University, Uppsala, Sweden; 4 Department of Pharmaceutical Analytical Chemistry, Faculty of Pharmacy, Al-Azhar University, Assiut Branch, Assiut, 71524 Egypt

**Keywords:** Biotechnology, Immunology, Environmental sciences, Chemistry, Analytical chemistry, Electrochemistry

## Abstract

Pathogenic bacteria cause disease outbreaks and threaten human health, prompting the research on advanced detection assays. Herein, we developed a selective molecular imprinted aptasensor for sensitive and prompt quantitation of *Staphylococcus aureus* (*S. aureus*) bacteria. The aptasensor was constructed by immobilization of aptamer on gold nanoparticles modified magnetic nanoparticles (apt-AuNPs@ Fe_3_O_4_). A functional monomer (o-phenylenediamine, o-phen) was electro-polymerized on the surface of the as-synthesized nanocomposite in the presence of a template (*S. aureus*). After removing *S. aureus*, the formed imprinted sites were available to extract pathogenic bacteria from complicated matrices. The surface morphology of the as-fabricated nanocomposites was characterized using different spectroscopic and electrochemical methods. Moreover, we thoroughly evaluated factors affecting the synthesis and determination procedures. The molecular imprinted aptasensor exhibited a wide linear range of 10^1^–10^7^ CFU mL^−1^ with a Limit of Detection, LOD (signal to noise = 3) of 1 CFU mL^−1^. The aptasensor detected *S. aureus* in milk, conduit water, and apple juice samples with good recoveries % and satisfactory relative standard deviations (RSDs %) values.

## Introduction

A Gram-positive bacterium, staphylococcus aureus (S. aureus) is an extremely significant food-borne pathogenic bacteria. Its harmful effects on humans include pneumonia, endocarditis, abscesses, and septicemia^1^. It was reported that *S. aureus* is highly infectious and causes some of the most common infections worldwide^2^. Moreover, it possesses multiple virulence factors, and thus, it can develop strong resistance to antibacterial agents^3^. Bacterial culture has been considered an ideal tool for detecting S. aureus. The main disadvantages of this technique are time-consuming, excessive overloads on the operator, and high demands on the laboratory environment^4^. Quantitative polymerase chain reaction (PCR) has attracted great attentions as it can decrease the analysis time and amplify the bacterial genome exponentially. However, polymerase enzyme can be inhibited by matrix-related factors such as a low count of target bacteria in large sample volumes^5^. Thus, it is vital to construct selective, sensitive, and cost-effective sensors to detect *S. aureus*. Electroanalytical techniques have drawn great attentions as a result of fast response, sufficient selectivity, low detection limits, simplicity, and low cost^6–12^.

Aptamer (Apt), a single-stranded DNA, has attracted much attentions as an alternative to antibodies due to its resistance to denaturation, ease of modification, and large scale chemical synthesis^13–15^. Upon binding to the target molecule, it can be folded into a unique 3D-conformation^16^. Molecular imprinted technology (MIT) was used to prevent the interference of very similar structures in different matrices. It was formed by the electro-polymerization of the functional monomer around the template. Specific binding sites are formed after the removal of the template, capable of identifying the analyte under study^17–20^. Interestingly, we found only one report in the literature describing the electrochemical sensing of *Pseudomonas aeruginosa* based on the fabrication of molecular imprinted aptasensor immobilized on gold nanoparticles/polydopamine hybrid^21^.

Magnetic nanoparticles (Fe_3_O_4_ NPs) have been given a lot of interests due to its excellent electro-catalytic properties, low cost, low toxicity, and super paramagnetic properties^22,23^. In addition, noble metals e.g. AuNPs are used to modify electrodes to increase the conductivity and enhance the electron transfer^24,25^.

Herein, a molecular imprinted aptasensor was prepared for selective and sensitive detection of *S. aureus*. The aptasensor is based on the modification of magnetic nanoparticles with gold nanoparticles (AuNPs@Fe_3_O_4_). The molecular imprinted polymer film was formed around AuNPs@Fe_3_O_4_ in the presence of *S. aureus* and apt via electro-polymerization of o-phen monomer. After removing the *S. aureus*, an apt-MIP is made accessible for *S. aureus* influx. The as-fabricated aptasensor was applied efficiently to determine *S. aureus* in water, milk, and apple juice samples. To the best of our knowledge, this is the first report that use Apt-MIP for targeting *S. aureus*.

## Experimental

### Materials, reagents, and instruments

Details for descriptions of materials, reagents, and instruments were listed in Electronic Supplementary Materials (ESM).

### Synthesis of magnetic nanoparticles (Fe_3_O_4_ NPs)

According to our previous work^22^, the magnetic nanoparticles were prepared with slight modifications. Briefly, 2.85 g FeCl_3_⋅6H_2_O and 1.24 g FeCl_2_⋅4H_2_O were sonicated in 35 mL DDW for 15 min until complete solubility. After that, 45 mL 1.5 M NaOH was added portion wise with continual stirring for another 15 min. The obtained black precipitate was washed with DDW and 15 mL HClO_4_. Then, the precipitate was dried in an oven at 37 °C for 3 h before washing four times with DDW. Finally, the black product was dried at 60 °C overnight and then ground and stored at 4 °C.

### Activation and preparation of aptamer (apt)

The apt was firstly activated at 90 °C for 15 min. After that, 150 μL of 2.5 μM apt in TBST buffer. Then, it was incubated with 10^7^ CFU mL^−1^ of *S. aureus* for 45 min at 37 °C.

### Preparation of AuNPs@Fe_3_O_4_/GCE

The GCE was polished until a shiny appearance using alumina slurry, methanol, and DDW. A volume of 5 μL Fe_3_O_4_ (5.0 mg mL^−1^) dispersed in ethanol was casted on the surface of GCE. After drying, gold nanoparticles (AuNPs) were electrodeposited on the surface of Fe_3_O_4_/GCE by immersion in 0.5 M Na_2_SO_4_ containing 1.5 mM HAuCl_4_ solution under a constant potential of − 0.2 V for 300 s.

### Fabrication of MIP-apt-AuNPs@Fe_3_O_4_/GCE

**Figure 1:**
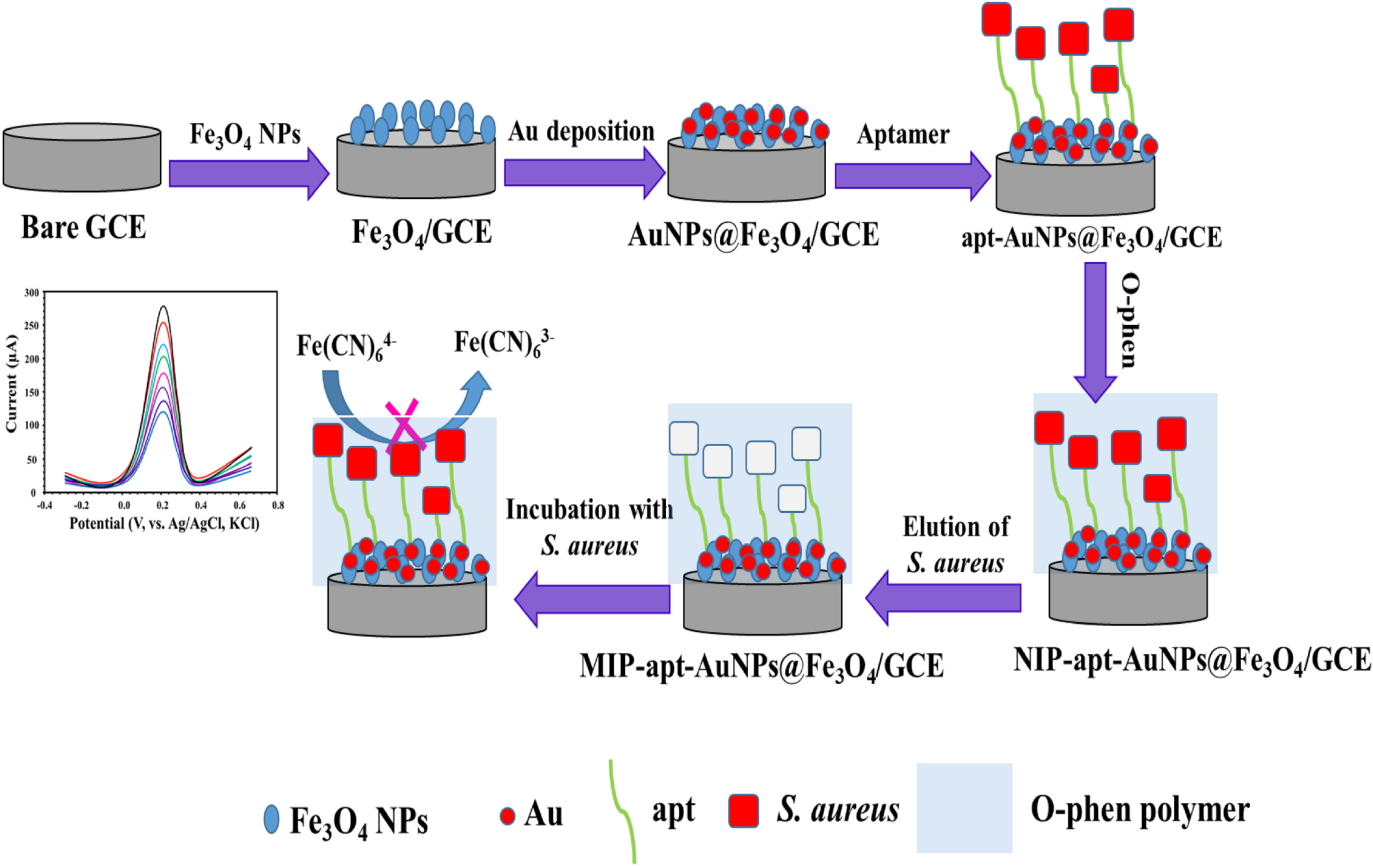
Representative diagram for the fabrication of the molecularly imprinted based aptasensor and its use for the determination of *S. aureus*.

Firstly, 10.0 μL of the *S. aureus*-apt complex (prepared in “Activation and preparation of aptamer (apt)”) was dropped on the surface of the AuNPs/Fe_3_O_4_/GCE where apt was covalently bound to AuNPs by strong Au–S bond. Then, Then, 15 μL of the *S. aureus* was cast on the electrode's surface to impregnate any free apt. Secondly, 1.45 mM o-phen was electro-polymerized on the surface of the electrode by sweeping the potential in the range of -0.4–0.9 V using a scan rate 100 mV s^−1^ for 15 cycles. Finally, the electrode was placed in a solution containing 0.01 M SDS and 7% HNO_3_ (dissolved in DDW) for 60 min to remove *S. aureus* from its imprinted sites. A non-imprinted polymer (NIP) was prepared using the same steps without adding the template (*S. aureus*) (Fig. 1).

### Preparation of real samples

The milk sample (0.5 mL) was mixed with 1.5 mL acetonitrile and spiked with *S. aureus* before shaking for 30 s. The mixture was sonicated for 5 min and then centrifuged at 3000 rpm for 10 min, and the supernatant was collected for further analysis. Drug-free milk samples were prepared using the same steps without spiking with *S. aureus*^26^. Conduit water was filtered to remove the insoluble and floated matters and stored in high-quality clean polyethylene containers. Water samples (5.0 mL) were spiked with different concentrations of *S. aureus* and stored at − 4 °C until analysis^22,27,28^. Apple juice samples obtained from the local market were analyzed without any further treatment.

## Results and discussions

### Characterization of nanocomposites

Scanning Electron Microscope (SEM) was used to check the different synthesized nanomaterials as depicted in Fig.S1. Magnetic nanoparticles (Fe_3_O_4_ NPs) are uniformly distributed with an average size of 14.5 nm (Fig. S1A). After modification with AuNPs, the size was increased to 38.5 nm, suggesting the successful decoration of Fe_3_O_4_ NPs with AuNPs (Fig. S1B). Functional monomer (o-phen) was electro-polymerized on the surface of AuNPs@Fe_3_O_4_/GCE in the presence of template (*S. aureus*) and thiolated apt, resulting in the complete coverage with a film of o-phen polymer (NIP) (Fig. S1C). After the removal of *S. aureus* from its imprinted sites, narrow pores were formed on the surface of polymer network resulting in the formation of molecular imprinted polymer (MIP) film (Fig. S1D). Fig.S2 shows the energy dispersive X-ray spectroscopy (EDX) of AuNPs@Fe_3_O_4_ nanocomposite with the main elements of O, Fe, and Au. Fig.S3 exhibits the FTIR spectra of Fe_3_O_4_ (green), AuNPs@Fe_3_O_4_ (red), and apt-AuNPs@Fe_3_O_4_ (black) where absorption bands at 3420, 1705, 1470, and 615 cm^−1^ are ascribed to υ (OH) of surface adsorbed water, υ (C=O), δ (OH), and υ (Fe–O), respectively^22^. In addition, Fig. S4 shows the Zeta potentials of the as-synthesized nanocomposites including Fe_3_O_4_, AuNPs/Fe_3_O_4_, and apt- AuNPs/Fe_3_O_4_. The obtained Zeta potential values of Fe_3_O_4_, AuNPs/Fe_3_O_4_, and apt- AuNPs/Fe_3_O_4_ were found to be 32.8 ± 0.56, 22.3 ± 1.09, and 10.23 ± 1.57, respectively. It is seen that the potential values decrease after coupling of positively charged Fe_3_O_4_ with AuNPs followed by further decrease upon binding with negatively charged apt, suggesting the successful formation of apt- AuNPs/Fe_3_O_4_.

### Electrochemical characterization of the as-synthesized nanocomposites

**Figure 2:**
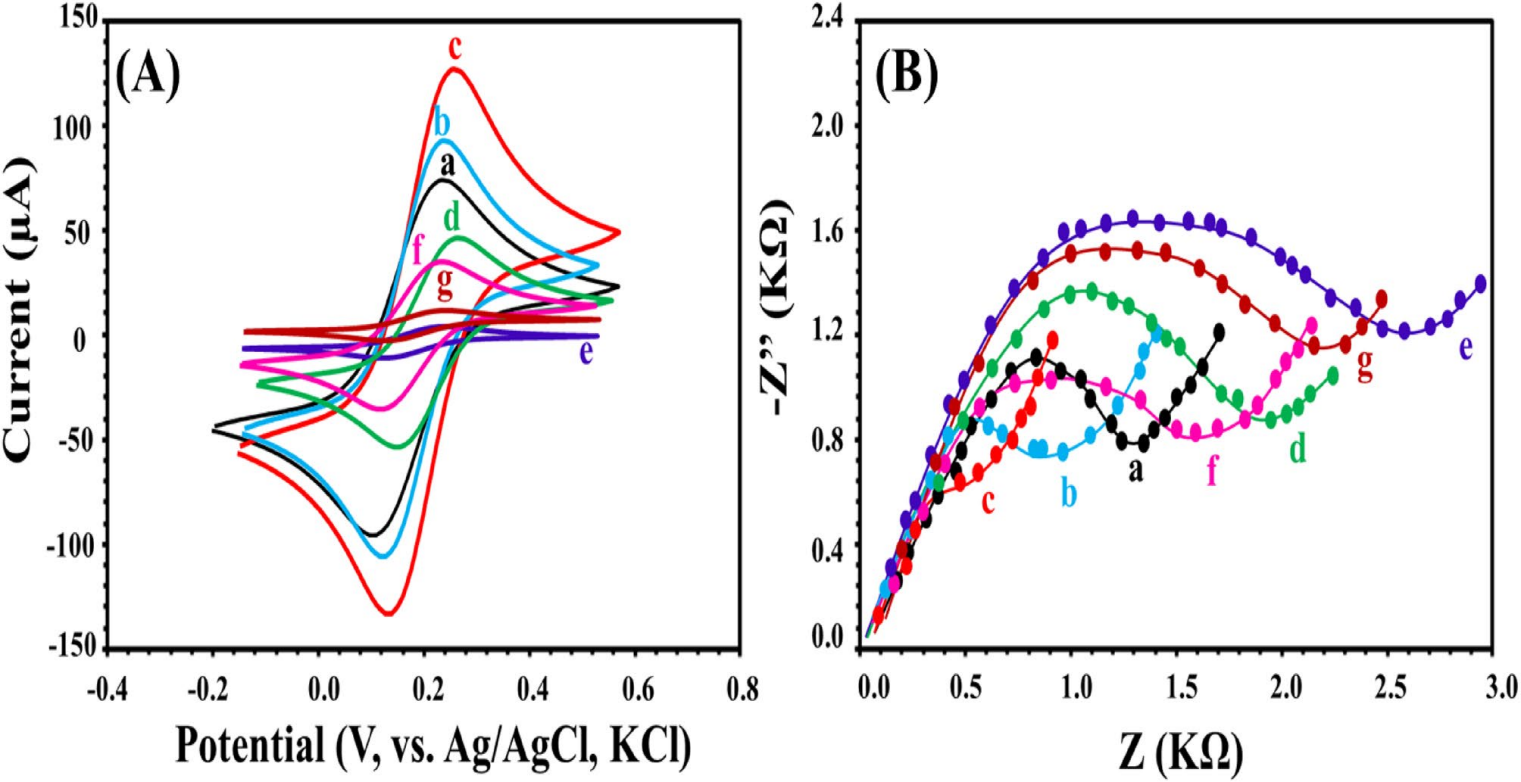
CV (**A**) and EIS (**B**) of bare GCE (a), Fe_3_O_4_/GCE (b), AuNPs@ Fe_3_O_4_/GCE (c), apt-AuNPs@ Fe_3_O_4_/GCE (d), NIP-apt-AuNPs@ Fe_3_O_4_/GCE (e), MIP-apt-AuNPs@ Fe_3_O_4_/GCE (f), and MIP-apt-AuNPs@ Fe_3_O_4_/GCE after rebinding of *S. aureus* (g). Conditions are 5.0 mM Fe(CN)_6_^3-/4-^ dissolved in 0.1 M KCl.

Different electrodes were prepared and evaluated using cyclic voltammetry (CV) and electrochemical impedance spectroscopy (EIS). They were immersed in a solution of 5.0 mM Fe(CN)_6_^3-/4-^ dissolved in 0.1 M KCl. Figure 2A_a_ exhibits the redox peaks of Fe(CN)_6_^3-/4-^ at bare GCE where it shows to identifiable and separated anodic and cathodic peaks. After modification with Fe_3_O_4_ NPs, the redox currents of the redox probe were increased as a result of enhanced surface area and good conductivity of Fe_3_O_4_ NPs (Fig. 2Ab). Further enhancement of the redox currents was observed after modification with AuNPs (Fig. 2Ac), suggesting the excellent conductivity of AuNPs. Attachment of apt to the surface of AuNPs@ Fe_3_O_4_/GCE resulted in the decrease of the peak currents of Fe(CN)_6_^3-/4-^ due to the repulsion between the negatively charged apt and negatively charged redox probe (Fig. 2Ad). Electro-polymerization of o-phen monomer on the surface of apt- AuNPs@ Fe_3_O_4_/GCE and in the presence of *S. aureus* i.e. NIP- apt- AuNPs@ Fe_3_O_4_/GCE, the peak currents of Fe(CN)_6_^3-/4-^ were sharply deceased due to the formation of insulating layer that inhibited the influx of the redox probe (Fig. 2Ae). The removal of *S. aureus* from its imprinted sites i.e. MIP- apt- AuNPs@ Fe_3_O_4_/GCE increased the peak currents of Fe(CN)_6_^3-/4-^ due to the creation of numerous imprinted sites for the flowing of the redox probe, but it is still lower than apt- AuNPs@ Fe_3_O_4_/GCE (Fig. 2Af). After rebinding the *S. aureus*, the peak currents of the redox probe were dramatically decreased as the cavities of the imprinted layers were relocked by the template (Fig. 2Ag). Moreover, the electrochemical activities of different interfaces were demonstrated using EIS (Fig. 2B). It is shown that the semicircle diameter was changed after each modification.

### Optimization of experimental conditions

Optimizations of incubation time, pH effect, elution time, deposition potential and time of AuNPs, and apt concentration were listed in ESM.

### Analytical figures of merit

**Figure 3:**
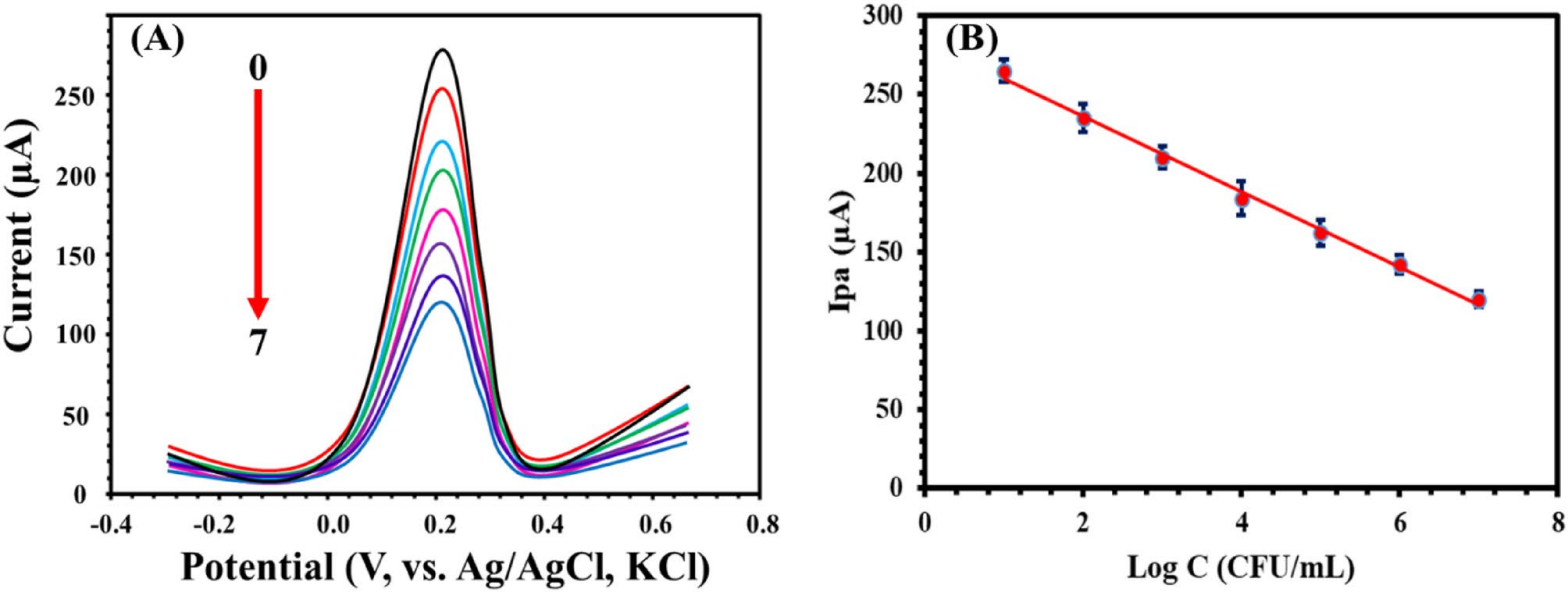
(**A**) DPVs of MIP-apt-AuNPs@ Fe_3_O_4_/GCE after incubation with different concentrations of the *S. aureus* (0:0, 1:10^1^, 2:10^2^, 3:10^3^, 4: 10^4^, 5:10^5^, 6: 10^6^ and 7: 10^7^ CFU·mL^−1^ *S. aureus* in 0.1 M KCl containing 5.0 mM [Fe(CN)_6_]^3−/4−^ and (**B**) is a calibration plot. Conditions of DPV are pulse height of 30 mV, pulse width of 0.08 s and step height of 15 mV.

The sensitivity of the proposed aptasensor towards *S. aureus* was measured using differential pulse voltammetry (DPV) under optimized conditions. Figure 3A shows that the peak currents of the redox probe at MIP-apt-AuNPs@ Fe_3_O_4_/GCE were decreased after the increase in the concentration of *S. aureus*. The calibration plot shown in Fig. 3B was linear over the range of 10^1^–10^7^ CFU mL^-1^ with a linear regression of Ipa (µA) = − 23.9 Log C_S. aureus_ + 283.9 (R^2^ = 0.9986). According to IUPAC recommendation (IUPAC 1976), the analyte’s signal at the detection limit (S_dl_) is given by:

$$ {\text{S}}_{{{\text{dl}}}} = {\text{ S}}_{{{\text{reag}}}} + {\text{ k }}* \, \sigma_{{{\text{reag}},}} $$

where S_reag_ is the electrochemical signal for a blank, σreag is the known standard deviation for the blank’s electrochemical signal (n = 10). As is well known, k = signal/noise (S/N) = 3. As suggested by Long and Winefordner (1983) (Long and Winefordner 1983), the use of k = 3 allows a confidence level of 99.86% for a normal distribution of the blank signals. The detection limit can be calculated by S_dl_ and calibration curves. The LOD was calculated as 1 CFU mL^−1^. Moreover, the method with compared with other reported methods for the determination of S. aureus (Table 1). I t was found that the proposed aptasensor exhibits wide-linear range and low detection value.

**Table 1:** Comparison between the proposed aptasensor and other reported sensors for the determination of *S. aureus.*

Sensor	Method	Linear range (CFU mL^−1^)	LOD (CFU mL^−1^)	Reference
Apt/single walled carbon nanotubes	Potentiometry	10^3^–10^8^	8 × 10^2^	^29^
Apt/reduced graphene oxide/AuNPs	Impedimetry	10^1^–10^6^	10	^30^
Apt/Au electrode	Impedimetry	10^1^–10^4^	10	^31^
Apt/S. aureus/apt-AgNP sandwich complex	DPV	10^1^–10^6^	1	^32^
Apt/AuNPs/carbon nanoparticles/cellulose nanofibers	Impedimetry	1.2 × 10^1^–1.2 × 10^8^	1	^33^
**MIP-apt-AuNPs@ Fe**_**3**_**O**_**4**_**/GCE**	**DPV**	**10**^**1**^**–10**^**7**^	**1**	**This work**

### Reproducibility, repeatability, and stability of MIP-apt-AuNPs@ Fe_3_O_4_/GCE

**Figure 4:**
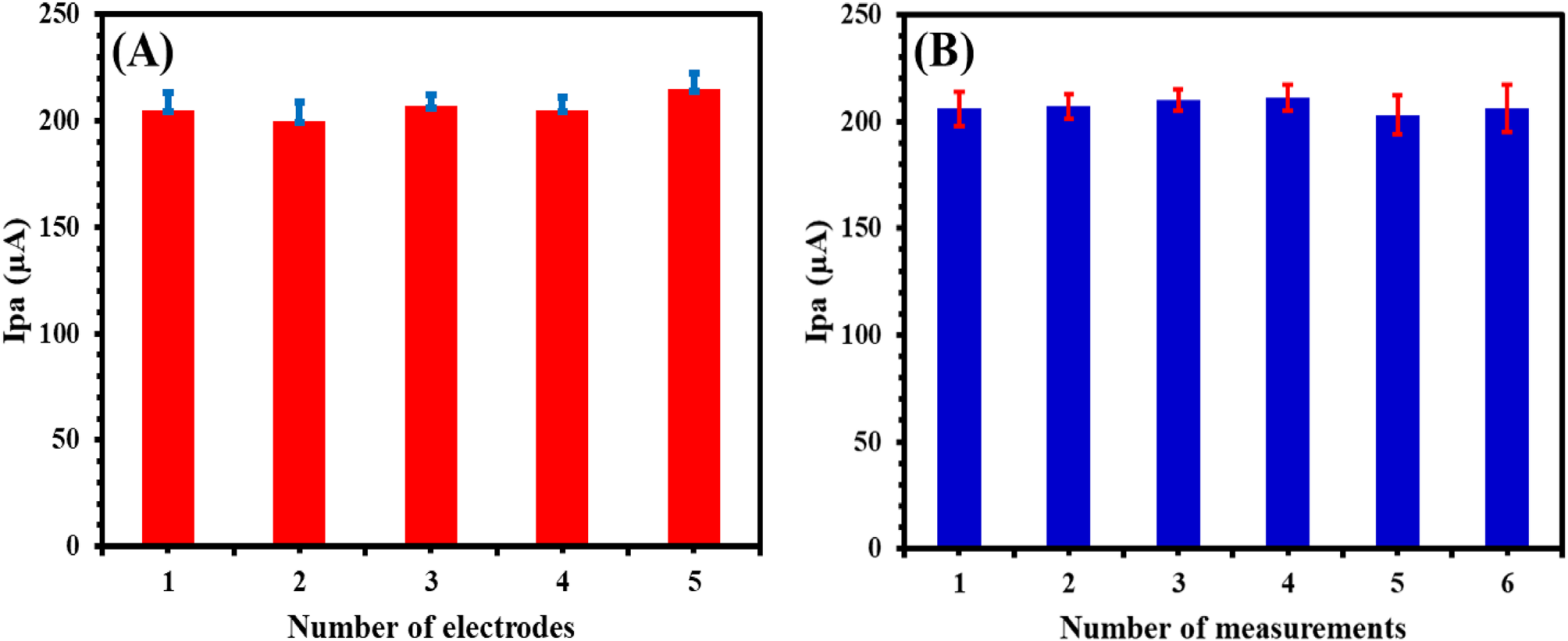
Reproducibility (**A**) and repeatability (**B**) of MIP-apt-AuNPs@ Fe_3_O_4_/GCE after incubation with of the 10^3^ CFU mL^-1^ *S. aureus* in 0.1 M KCl containing 5.0 mM [Fe(CN)_6_]^3−/4−^. Conditions of DPV are pulse height of 30 mV, pulse width of 0.08 s and step height of 15 mV.

Reproducibility was measured by monitoring the DPV responses of five fabricated aptasensor prepared under the same conditions (Fig. 4A). It was found that the relative standard deviation % (RSD %) did not exceed 3.2%. Moreover, the repeatability was measured via measuring the DPV responses for six readings and calculating the RSD % that did not exceed 2.6% (Fig. 4B).

Moreover, the stability of the aptasensor was examined using CV at scan rate of 300 mV s^−1^ in 0.1 M phosphate buffer and 5.0 mM [Fe(CN)_6_]^3−/4−^. After 50 cycles, DPV readings did not appreciably change, confirming the excellent stability of the proposed sensor (Fig. S10A). In addition, the long term stability of the aptasensor was studied by storing the aptasensor at 4 °C and it was used to detect *S. aureus* every week for one month (Fig.S10 B). It was found that the proposed aptasensor retained about 95% of its original activity for four weeks.

### Specificity of MIP-apt-AuNPs@ Fe_3_O_4_/GCE

**Figure 5:**
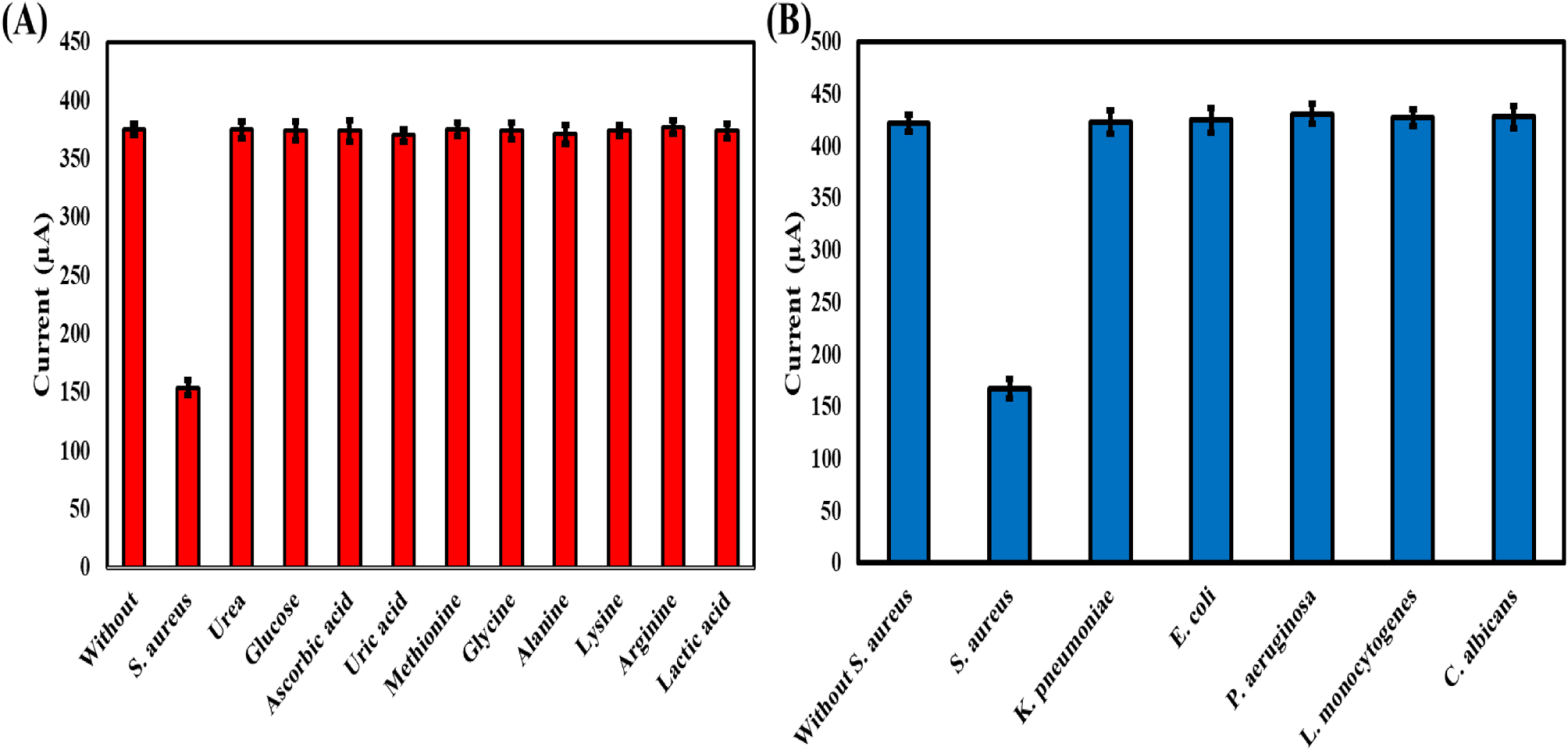
(**A**) CV scans of MIP-apt-AuNPs@ Fe_3_O_4_/GCE in presence of 10^3^ CFU mL^−1^ *S. aureus* and 300 µM some common organic compounds in 0.1 M phosphate buffer containing 5.0 mM [Fe(CN)_6_]^3−/4−^ at ν = 300 mV s^−1^. (B) CV scans of MIP-apt-AuNPs@ Fe_3_O_4_/GCE in presence of 10^3^ CFU mL^−1^ *S. aureus* and 10^6^ CFU mL^−1^ interfering bacteria in 0.1 M phosphate buffer containing 5.0 mM [Fe(CN)_6_]^3−/4−^ at ν = 300 mV s^−1^.

The specificity of the as-fabricated aptasensor was evaluated by immersing in 10^3^ CFU contained in 300 µM organic compounds such as urea, glucose, ascorbic acid, uric acid, methionine, glycine, alanine, lysine, arginine, and lactic acid. Moreover, CV responses of the as-prepared aptasensor were recorded in the presence of 10^6^ CFU mL^−1^ *Klebsiella pneumonia (K. pneumonia)*, *Escherichia col*i (*E. col*i,), *Pseudomonas aeruginosa (P. aeruginosa)*, *Listeria monocytogenes* (*L. monocytogenes*), and *Candida albicans (C. albicans)*. Figures 5 A&B show that slight variations in currents were observed after addition of interfering compounds and bacteria. Moreover, it is obvious that only *S. aureus* can decrease the currents of redox probe, which is attributed to the molecular imprinted spaces and apt are well fitted for *S. aureus*.

### Applications of MIP-apt-AuNPs@ Fe_3_O_4_/GCE

**Figure 6:**
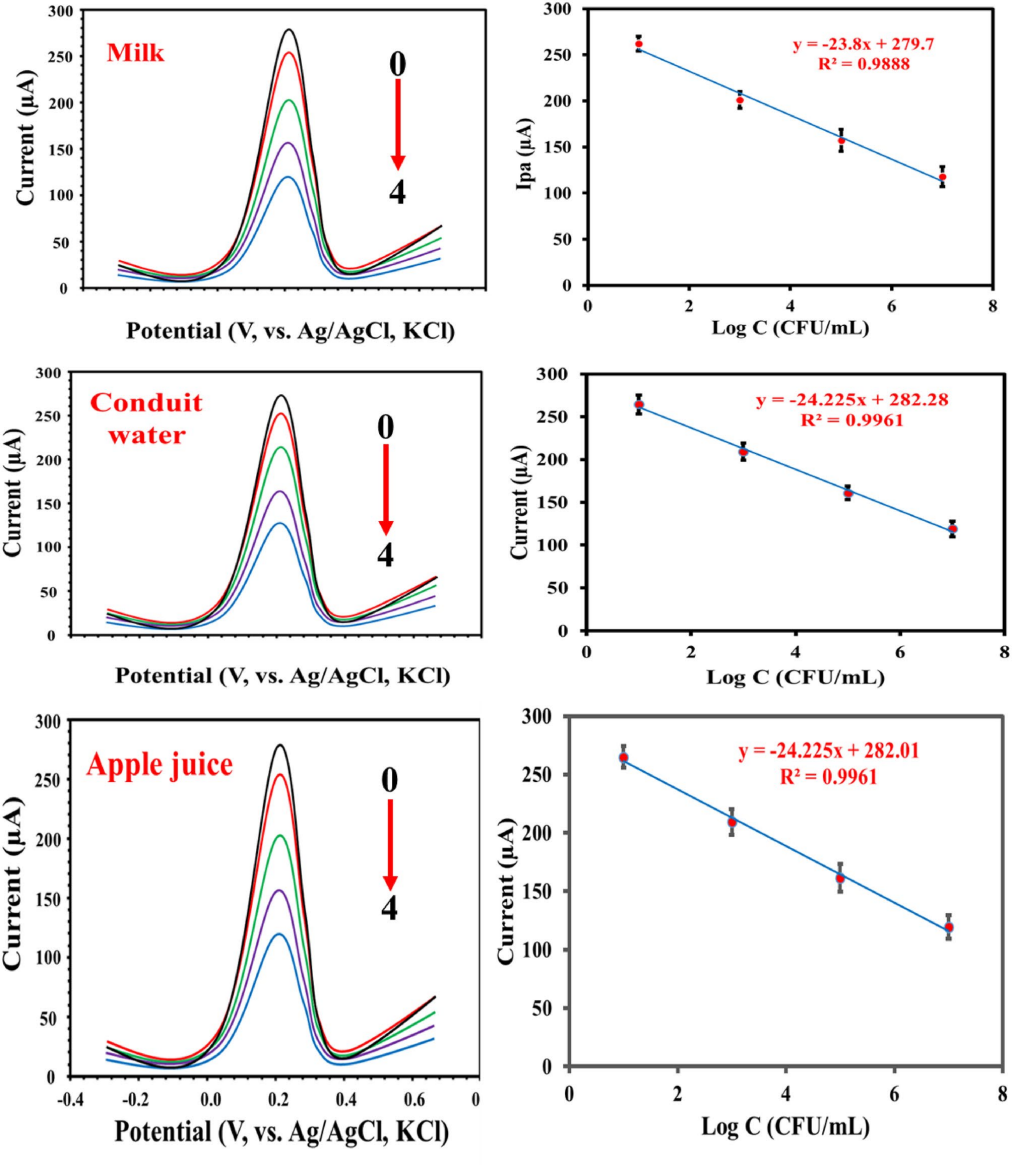
DPVs of MIP-apt-AuNPs@ Fe_3_O_4_/GCE after incubation with different concentrations of the *S. aureus* (0:0, 1:10^1^, 2:10^3^, 4:10^5^, and 4: 10^7^ CFU·mL^−1^ *S. aureus* in milk, conduit water, and apple juice samples. Conditions are concentration of [Fe(CN)_6_]^3−/4−^  = 5.0 mM, pulse height of 30 mV, pulse width of 0.08 s, and step height of 15 mV.

We tested the aptasensor for the detection of S. aureus in milk, conduit water, and apple juice samples. The concentration of *S. aureus* was adjusted to 0.5 McFarland turbidity. After that, the samples were spiked with different concentrations of *S. aureus*. Table 2 shows that the recoveries % ranged from 96 to 104% with relative standard deviations (RSDs) less than 3.4%, suggesting the suitability of the aptasensor for measuring *S. aureus* in milk, conduit water, and apple juice samples. Calibration plots for different artificial samples were shown in Fig. 6.

**Table 2:** Applications of MIP-apt-AuNPs@ Fe_3_O_4_/GCE for the determination of S. aureus in milk, conduit water, and apple juice samples.

Sample	Added (CFU mL^−1^)	Found (CFU mL^−1^)	Recovery % ± SD	RSD %
**Milk**				
1	10^2^ 10^4^	1.02 × 10^2^ 1.02 × 10^4^	102 ± 2.9 102 ± 3.4	2.8 3.3
2	10^2^ 10^4^	1.03 × 10^2^ 0.99 × 10^4^	103 ± 2.3 99 ± 2.7	2.2 2.7
3	10^2^ 10^4^	1.00 × 10^2^ 1.02 × 10^4^	100 ± 2.7 102 ± 3.4	2.7 3.3
**Conduit water**				
1	10^2^ 10^4^	1.01 × 10^2^ 0.98 × 10^4^	101 ± 2.3 98 ± 2.9	2.2 3.0
2	10^2^ 10^4^	0.99 × 10^2^ 0.96 × 10^4^	99 ± 3.0 96 ± 2.0	3.0 3.4
3	10^2^ 10^4^	1.03 × 10^2^ 1.04 × 10^4^	103 ± 3.5 104 ± 2.7	3.4 2.6
**Apple juice**				
1	10^2^ 10^4^	0.99 × 10^2^ 0.98 × 10^4^	99 ± 2.8 98 ± 3.0	2.8 3.1
2	10^2^ 10^4^	1.01 × 10^2^ 1.00 × 10^4^	101 ± 3.1 100 ± 2.7	3.1 2.7
3	10^2^ 10^4^	0.97 × 10^2^ 0.99 × 10^4^	97 ± 2.5 99 ± 2.8	2.6 2.8

## Conclusion

In this context, an ultrasensitive and selective molecular imprinted based aptasensor was fabricated to detect S. aureus. The aptasensor consists of gold nanoparticles modified magnetic nanoparticles loaded on the glassy carbon electrode surface (AuNPs@ Fe_3_O_4_/GCE). The thiolated aptamer was attached to the nanocomposite surface via Au–S covalent bond. A polymer film was deposited over the surface of the AuNPs@ Fe_3_O_4_/GCE by electro-polymerization of o-phen in the presence of the template (*S. aureus*). After elution of the template, the formed imprinted cavities were formed that can extract *S. aureus* from the complicated matrices. Simplicity, low LOD, good stability, low cost, high sensitivity, and high selectivity are the main advantages of the proposed aptasensor. The molecular imprinted aptasensor was applied efficiently for the detection of *S. aureus* in milk, conduit water, and apple juice samples.

## Supplementary Information

Supplementary Information.

## Data Availability

All data generated or analyzed during this study are included in this published article and its supplementary information files.
