# Supplementary material for: An innovative dual recognition aptasensor for specific detection of Staphylococcus aureus based on Au/Fe3O4 binary hybrid
Source: Sci Rep. 2022 Jul 22;12:12502. doi: 10.1038/s41598-022-15637-1 (PMC9307609; doi:10.1038/s41598-022-15637-1)
Supplement: Supplementary file 1 — Supplementary Information. [file 41598_2022_15637_MOESM1_ESM.docx]

**Supporting information**

**An innovative dual recognition aptasensor for specific detection of *Staphylococcus aureus* based on Au/ Fe_3_O_4_ binary hybrid**

**Mohamed M. El-Wekil^a^, Hamada Mohamed Halby^b^, Mahmoud Darweesh^b,c^, Mohamed E. Ali^b^, Ramadan Ali^d^**

^a^ Department of Pharmaceutical Analytical Chemistry, Faculty of Pharmacy, Assiut University, Assiut Branch 71524, Egypt

^b^ Department of Microbiology and Immunology, Faculty of Pharmacy, Al-Azhar University, Assiut Branch 71524, Egypt

^c^ Department of Medical Biochemistry and Microbiology, Uppsala University, Uppsala, Sweden

^d^ Department of Pharmaceutical Analytical Chemistry, Faculty of Pharmacy, Al-Azhar University, Assiut Branch 71524, Egypt

**Materials and reagents**

Gold (III) chloride trihydrate (HAuCl_4_^.^3H_2_O, 98 %) and o-phenylenediamine (o-phen, 98 %) were purchased from Sigma Aldrich, Germany. Ferric chloride hexahydrate (FeCl.6H_2_O, AR), ferrous chloride tetrahydrate (FeCl_2_.4H_2_O, AR), Hexadecyl trimethyl ammonium bromide (CTAB, AR), sodium hydroxide (NaOH, AR), sodium dodecyl sulfate (SDS, AR), perchloric acid (HClO_4_, AR), sodium dihydrogen phosphate (NaH_2_PO_4_, AR), and disodium hydrogen phosphate (Na_2_HPO_4_) were purchased from El- Nasser Co. for Intermediate Chemicals, Egypt. 5′ -thiol-modified DNA apt with the following sequence was used: 5′SH-GCA ATG GTA CGG TAC TTC CTC GGC ACG TTC TCA GTA GCG CTC GCT GGT CAT CCC ACA GCT ACG TCA AAA GTG CAC GCT ACT TTG CTA A-3′) [1]. *Staphylococcus aureus* ATCC 6538 (*S. aureus*), *Klebsiella pneumoniae* ATCC 10031 (*K. pneumoniae*), *Escherichia coli* O157: H7 ATCC 25922 (*E. coli*), *Pseudomonas aeruginosa* ATCC 27853 (*P. aeruginosa*), *Listeria monocytogenes* ATCC 19115 (*L. monocytogenes*), and *Candida albicans* ATCC 10231 (*C. albicans*) were provided by Department of Microbiology, Al Azhar University, Assiut Branch, Egypt. The concentrations of bacteria were determined on the standard plate count agar using the counting method. Inactivation of bacteria was carried out using 5 % HCHO overnight at 4°C, and washed 4 times with phosphate buffered saline solution (pH 7.4). The inactive bacteria suspension was diluted to different serial concentrations and stored at 4°C. Double distilled water (DDW) was used along the whole study. TBST buffer (10 mM KCl, 10 mM Tris-HCl, 10 mM MgCl_2_, pH = 7.4 and 0.05% Tween 20) was used to dilute aptamer (apt).

**Instrumentation**

Electrochemical experiments were performed using Princeton VersaSTAT MC (VersaSTAT 3, Model RE-1, Princeton Applied Research, AMETEK, USA), using platinum wire as the auxiliary electrode, Ag/AgCl, 3M KCl as the reference electrode, and a bare/ modified glassy carbon electrode (GCE) as working electrodes. Shimadzu 1601PC UV–Vis was used to record absorption spectra. Morphologies of the nanocomposites were investigated via Scanning Electron Microscope (SEM, JEOL, USA). Elemental analysis was determined using NEX QC+ QuantEZ. Nicolet™ iS™10 spectrometer was used to show the main functional groups on nanocomposites.


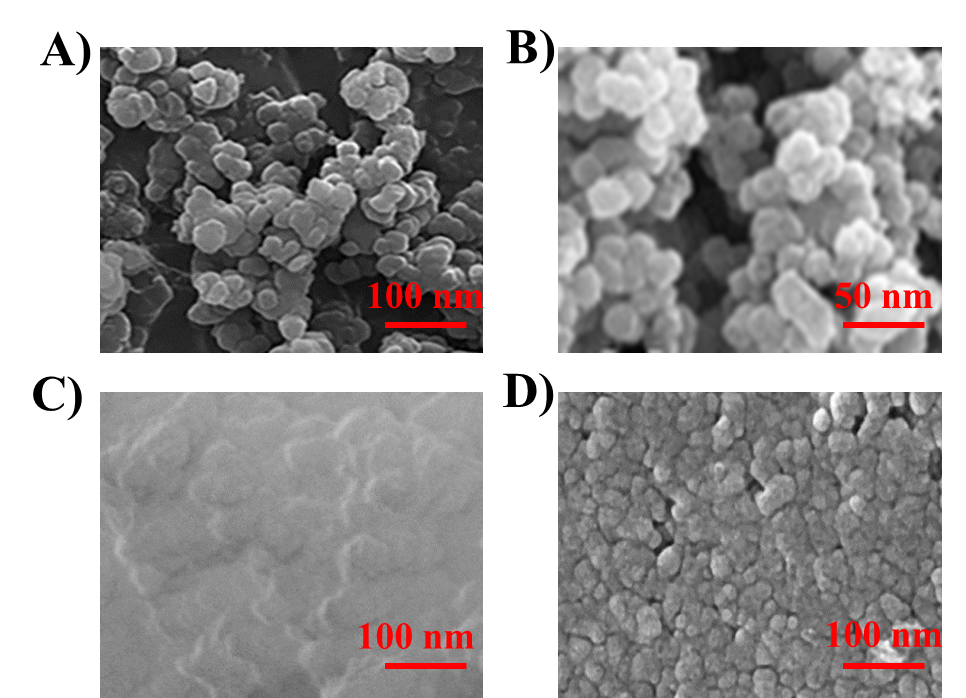


**Fig. S1** SEM images of Fe_3_O_4_/GCE (A), AuNPs/Fe_3_O_4_/GCE (B), NIP-apt-AuNPs@Fe_3_O_4_/GCE (C), and MIP-apt-AuNPs@Fe_3_O_4_/GCE (D).


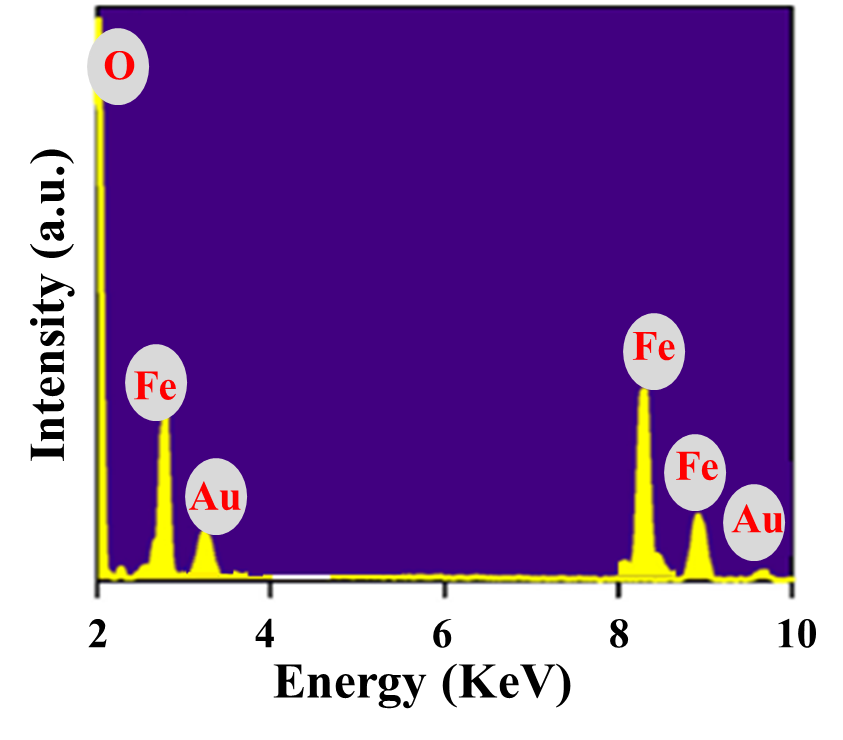


**Fig. S2** EDX of AuNPs/Fe_3_O_4_.


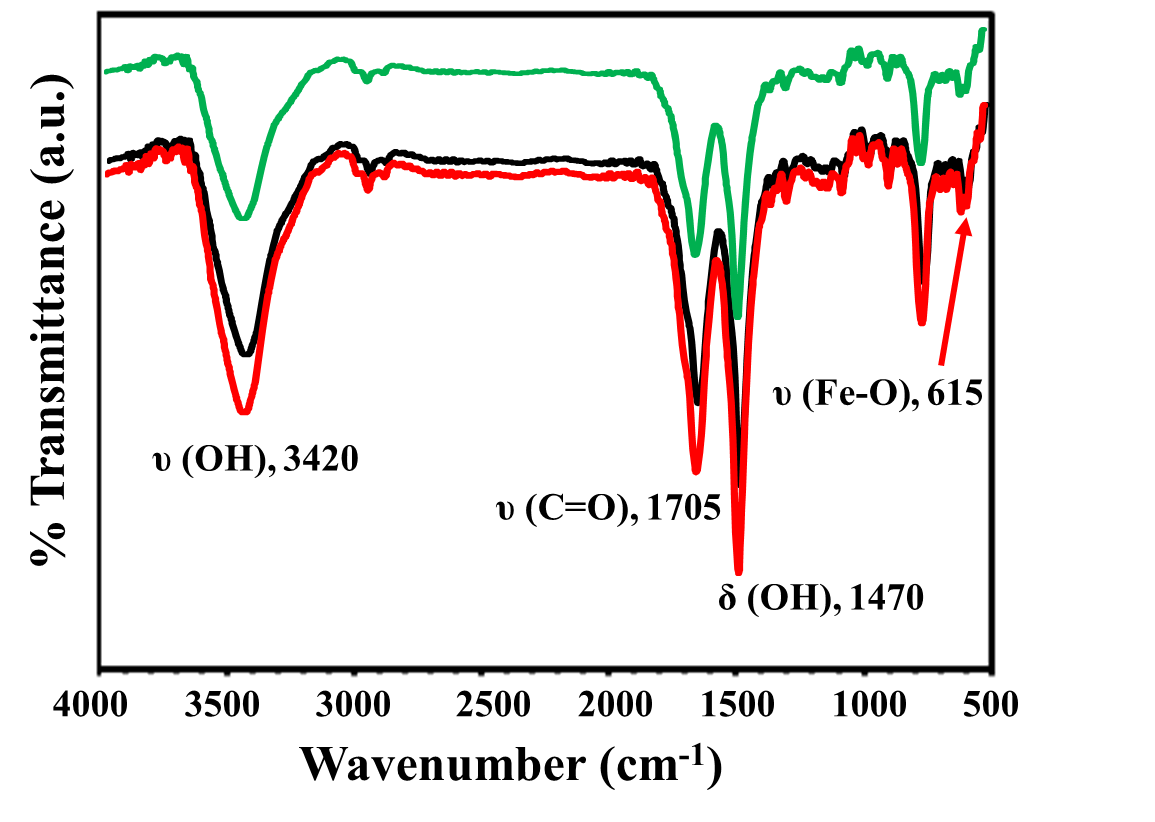


**Fig. S3** FTIR of Fe_3_O_4_ (green), AuNPs@Fe_3_O_4_ (red), and apt-AuNPs@Fe_3_O_4_ (black).


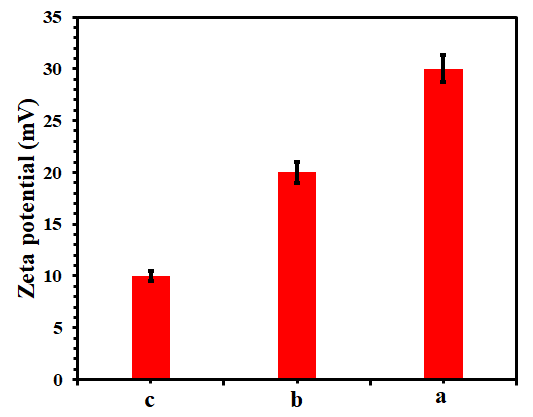


**Fig. S4** Zeta potentials of Fe_3_O_4_ (a), AuNPs@Fe_3_O_4_ (b), and apt-AuNPs@Fe_3_O_4_ (c).

**Optimization of experimental conditions**

**Incubation time**

To optimize the incubation time, the CVs of the proposed aptasensor in the absence and presence of *S. aureus* were recorded (dark and solid curves in all plots) in the solution containing 5.0 mM [Fe(CN)_6_]^3-/4-^ and 0.1 M phosphate buffer. Upon the addition of 10^3^ CFU mL^-1^ *S. aureus*, the change of anodic peak currents (∆Ipa) was enhanced as a function of incubation time (red and dashed curves in all plots). Based on Fig.S5, by increasing the incubation time from 15 min to 45, the change of anodic peak currents (∆Ipa) increases significantly and then start to level off. The maximum ΔIpa was achieved for 45 min incubation. As a result, so 45 min was chosen as optimum incubation time for subsequent experiments.

**Effect of pH**

In order to estimate the optimum pH value, a series of MIP-apt-AuNPs@ Fe_3_O_4_/GCE were incubated in the solution containing 10^3^ CFU mL^-1^ *S. aureus* for 45 min in different pH values. Fig.S6 shows that the maximum ΔIpa is recorded at pH = 7.0 in the solution of phosphate buffer containing 5.0 mM [Fe(CN)_6_]^3-/4-^. This could be explained by the effect of the strong acidic and alkaline solutions which influence the performance of apt, and further decrease the affinity between apt and the target molecule.

**Effect of elution time**

To construct a reproducible and selective electrochemical sensor, it is important to extract *S. aureus* from its imprinted sits. The effect of elution time was investigated via monitoring the change of anodic currents responses for different times. The results indicated that the maximum ΔIpa was achieved at 60 min and thereafter, increasing the elution time resulted in constant ΔIpa. Accordingly, we applied 60 min as an optimum elution time (Fig.S7).

**Effect of electro-deposition time of AuNPs**

The effect of electro-deposition time of AuNPs on the performance of MIP-apt-AuNPs@ Fe_3_O_4_/GCE was investigated (Fig.S8). Based on these results, with the increase of the deposition time from 50 to 400 s, the ΔIpa increased. When the deposition time increased to 350 s, the ΔIpa decreased, indicating that the size of nanoparticles increased and the electrochemical active area may be decreased. Thus, the optimum deposition time of 300 s was chosen.

**Apt concentration**

To optimize the concentration of the apt, various concentrations were prepared by adding different concentrations of apt onto MIP-apt-AuNPs@ Fe_3_O_4_/GCE surface. All the fabricated electrodes were then dipped in the solution containing equal amount of S. aureus. Fig.S9 shows the typical CV plots for various apt concentrations before and after *S. aureus* incubation. All the reads were recorded in the solution containing 5.0 mM [Fe(CN)_6_]^3-/4-^ and 0.1 M phosphate buffer. It was found that the ΔI_pa_ increases significantly by the increase of the apt concentration. When the concentration of apt is higher than 2.5 μM, the ΔI_pa_ intensity decreases by the rise of apt concentration, which could be attributed to the inactivation of some apt. The inactivation of apt can represent steric/conformational limitation and unfavorable interactions between neighboring apt such as cross hybridization. Based on the achieved results and data, 2.5 μM was chosen as optimized apt concentration throughout all experiments.

**
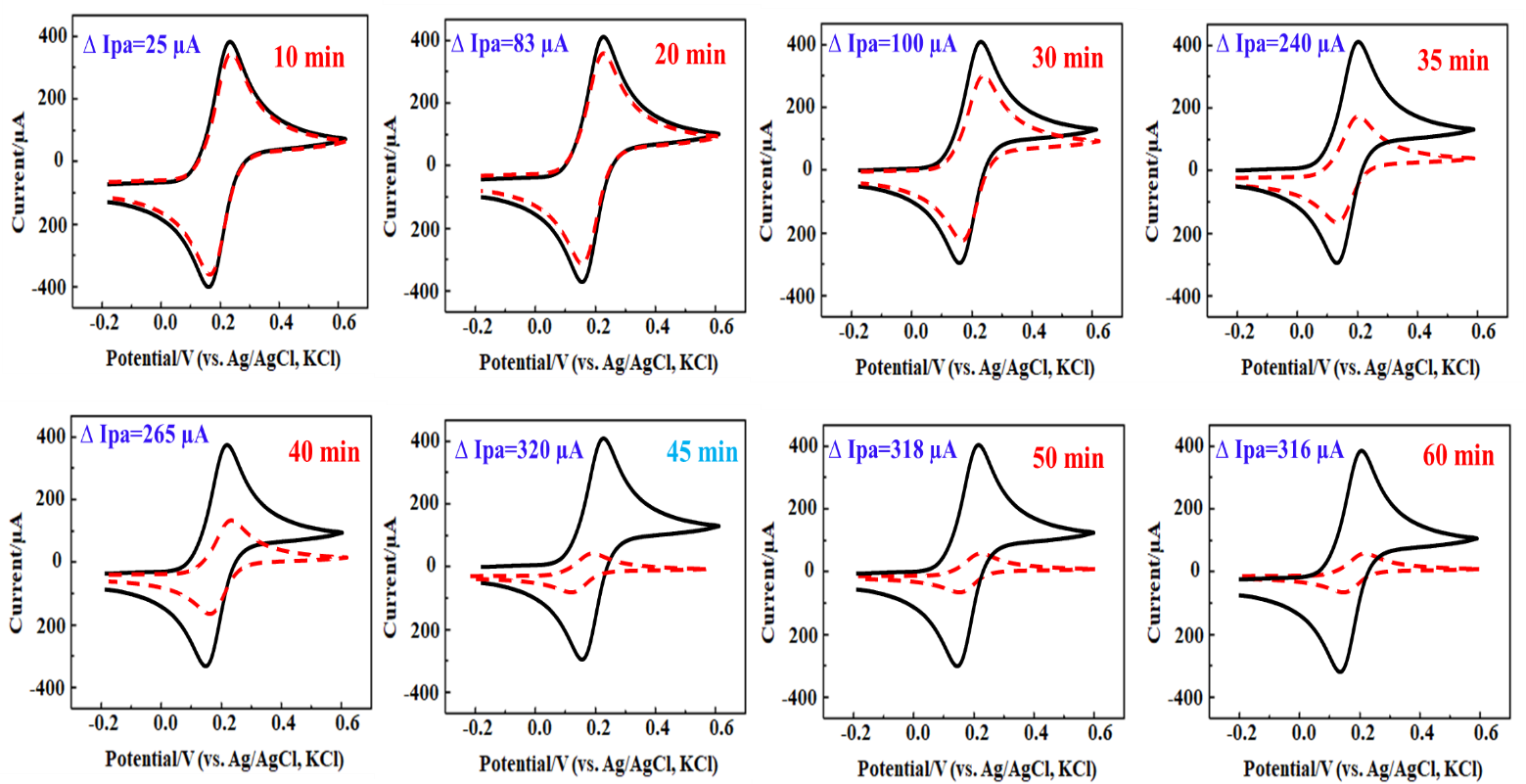
**

**Fig. S5** CVs of MIP-apt-AuNPs@ Fe_3_O_4_/GCE in the absence (dark and solid curves in all plots) and presence (red and dashed curves in all plots) of 10^3^ CFU mL^-1^ *S. aureus*, recorded in various incubation time. Conditions: redox electrolyte: 5.0 mM [Fe(CN)_6_]^3-/4-^ , 0.1 M phosphate buffer and scan rate = 300 mV s^-1^.

**
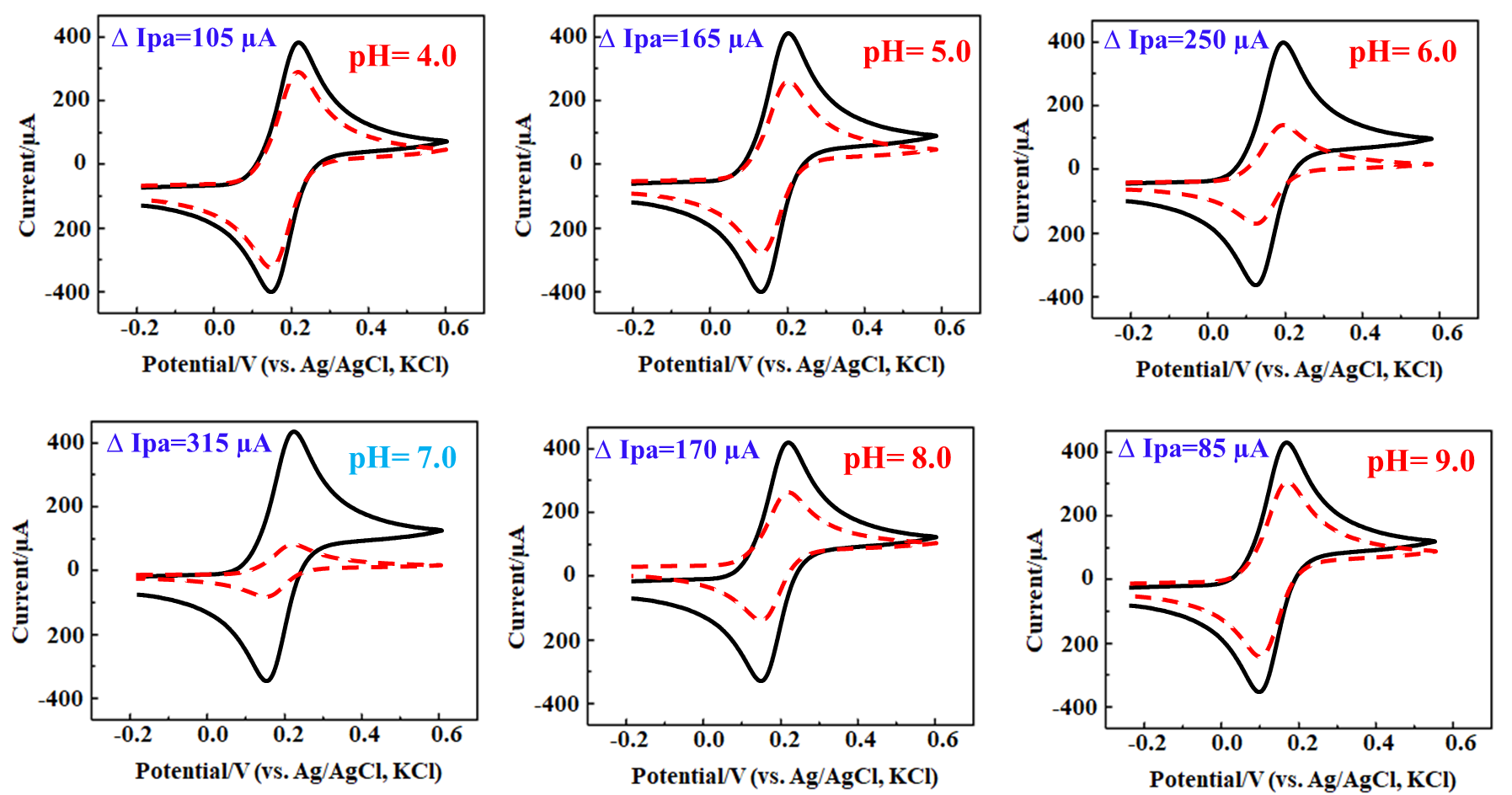
**

**Fig. S6** CVs of MIP-apt-AuNPs@ Fe_3_O_4_/GCE in the absence (dark and solid curves in all plots) and presence (red and dashed curves in all plots) of 10^3^ CFU mL^-1^ *S. aureus*, recorded in various pH values in 45 min. Conditions: redox electrolyte: Conditions: redox electrolyte: 5.0 mM [Fe(CN)_6_]^3-/4-^, 0.1 M phosphate buffer and scan rate = 300 mV s^-1^.

**
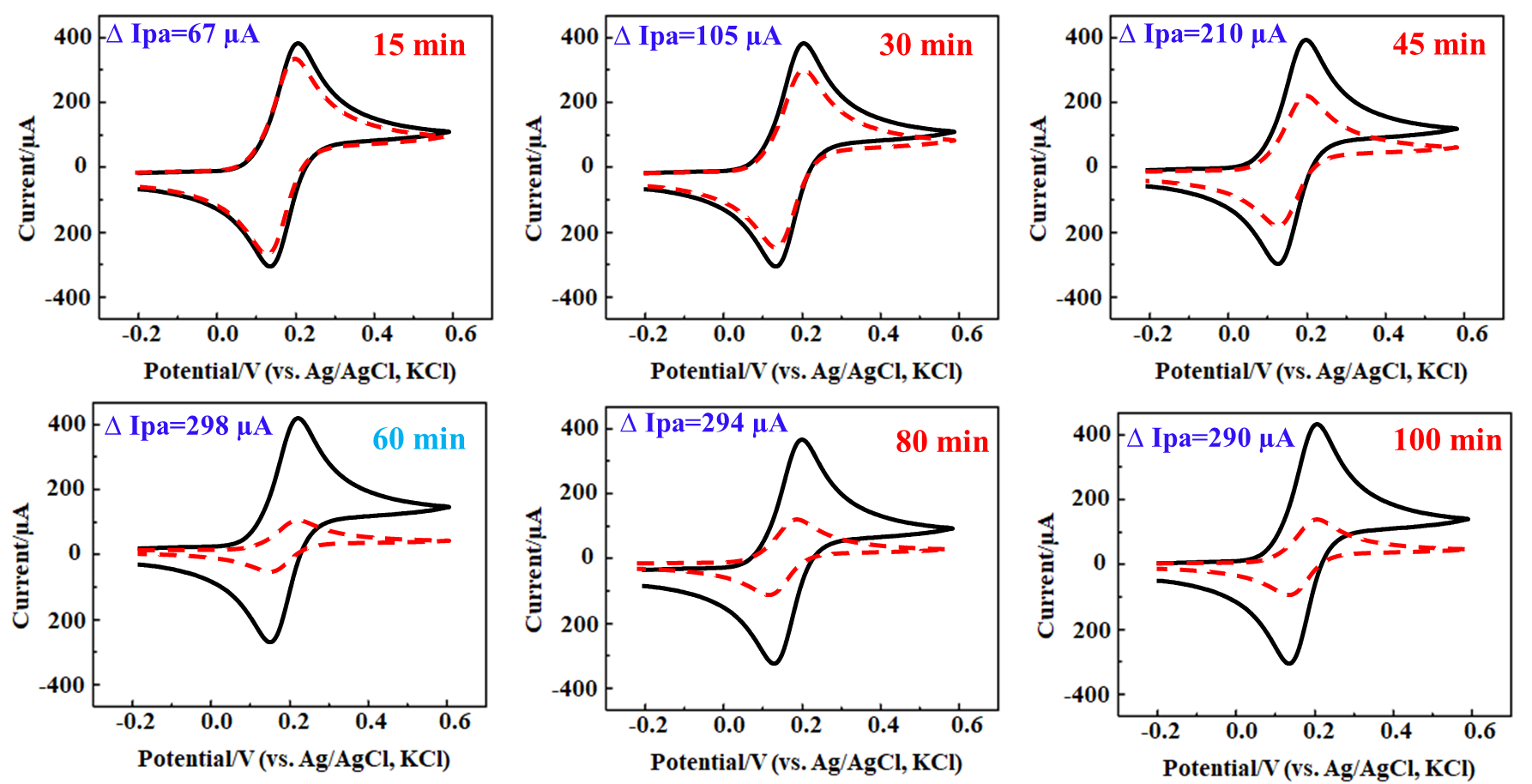
**

**Fig. S7** CVs of MIP-apt-AuNPs@ Fe_3_O_4_/GCE in the absence (dark and solid curves in all plots) and presence (red and dashed curves in all plots) of 10^3^ CFU mL^-1^ *S. aureus*, recorded after elution at different elution times. Conditions: Conditions: redox electrolyte: 5.0 mM [Fe(CN)_6_]^3-/4-^ , 0.1 M phosphate buffer and scan rate = 300 mV s^-1^.

**
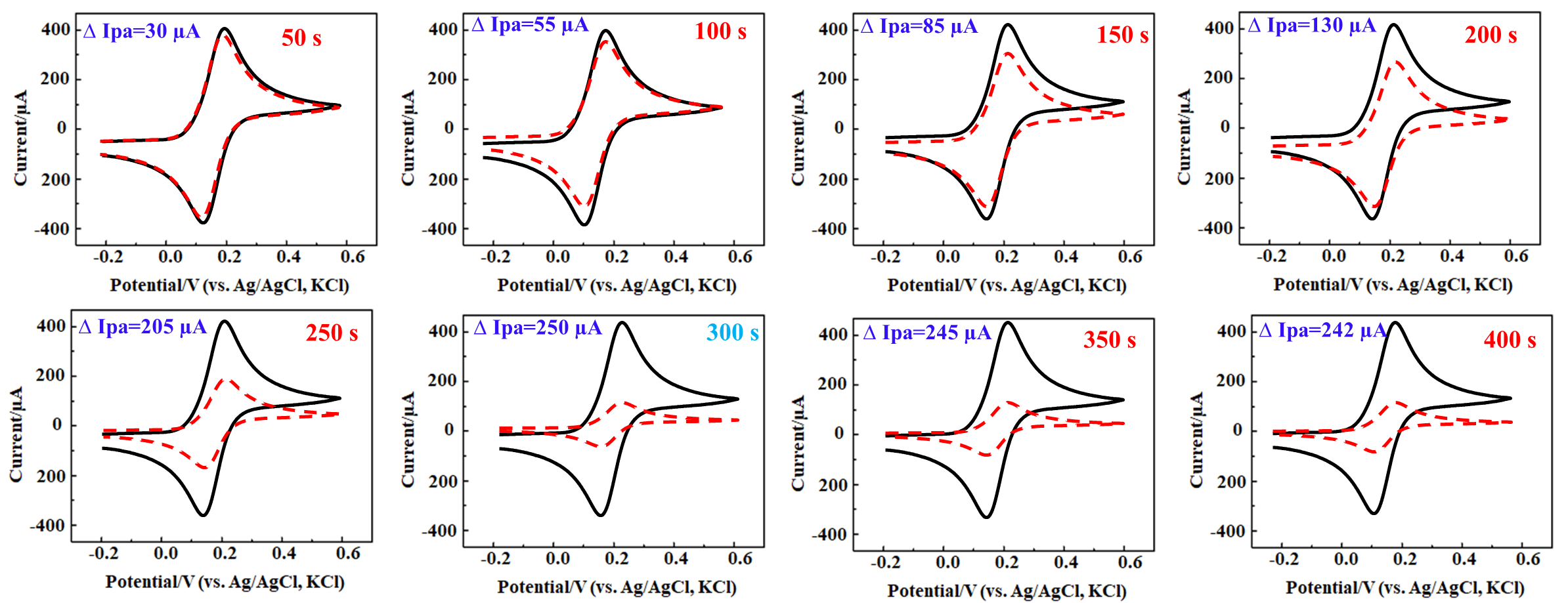
**

**Fig. S8** CVs of MIP-apt-AuNPs@ Fe_3_O_4_/GCE in the absence (dark and solid curves in all plots) and presence (red and dashed curves in all plots) of 10^3^ CFU mL^-1^ *S. aureus*, recorded after different electro-deposition times of AuNPs from 1.5 mM HAuCl_4_ solution. Conditions: Conditions: redox electrolyte: 5.0 mM [Fe(CN)_6_]^3-/4-^ , 0.1 M phosphate buffer and scan rate = 300 mV s^-1^.


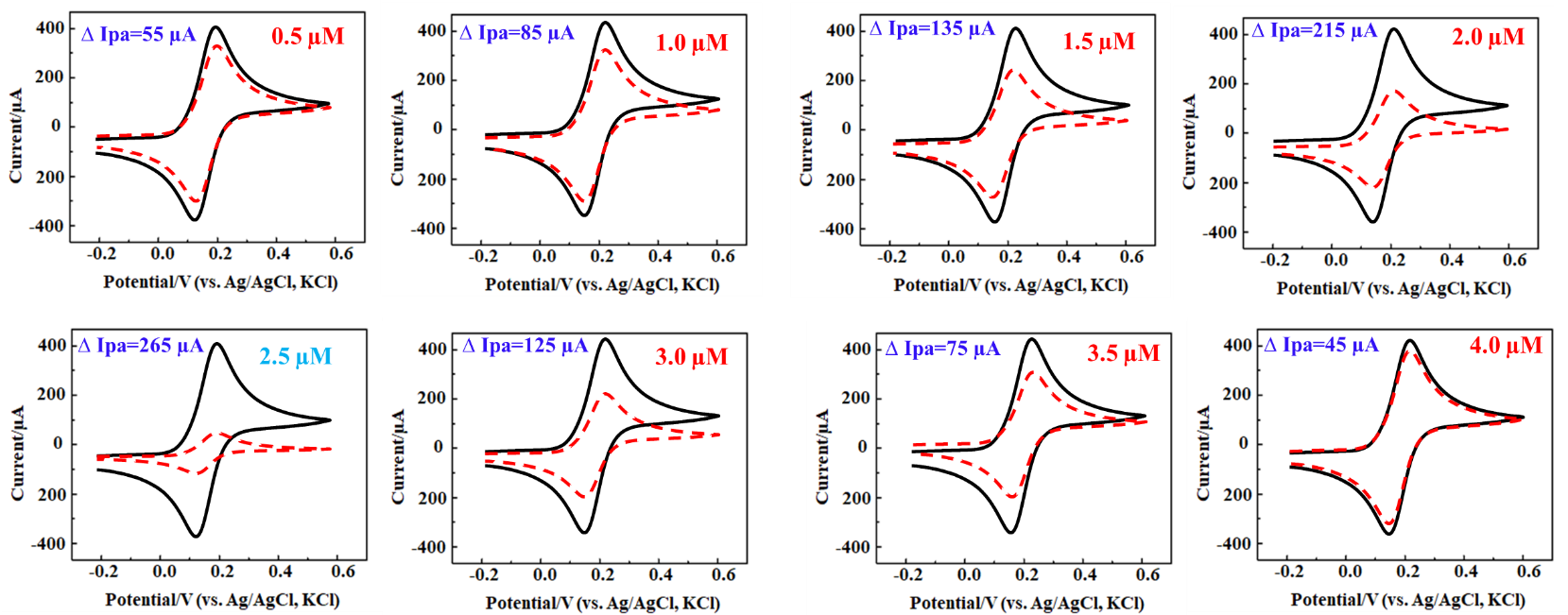


**Fig. S9** CVs of MIP-apt-AuNPs@ Fe_3_O_4_/GCE in the absence (dark and solid curves in all plots) and presence (red and dashed curves in all plots) of 10^3^ CFU mL^-1^ *S. aureus*, recorded after different apt concentrations. Conditions: Conditions: redox electrolyte: 5.0 mM [Fe(CN)_6_]^3-/4-^ , 0.1 M phosphate buffer and scan rate = 300 mV s^-1^.


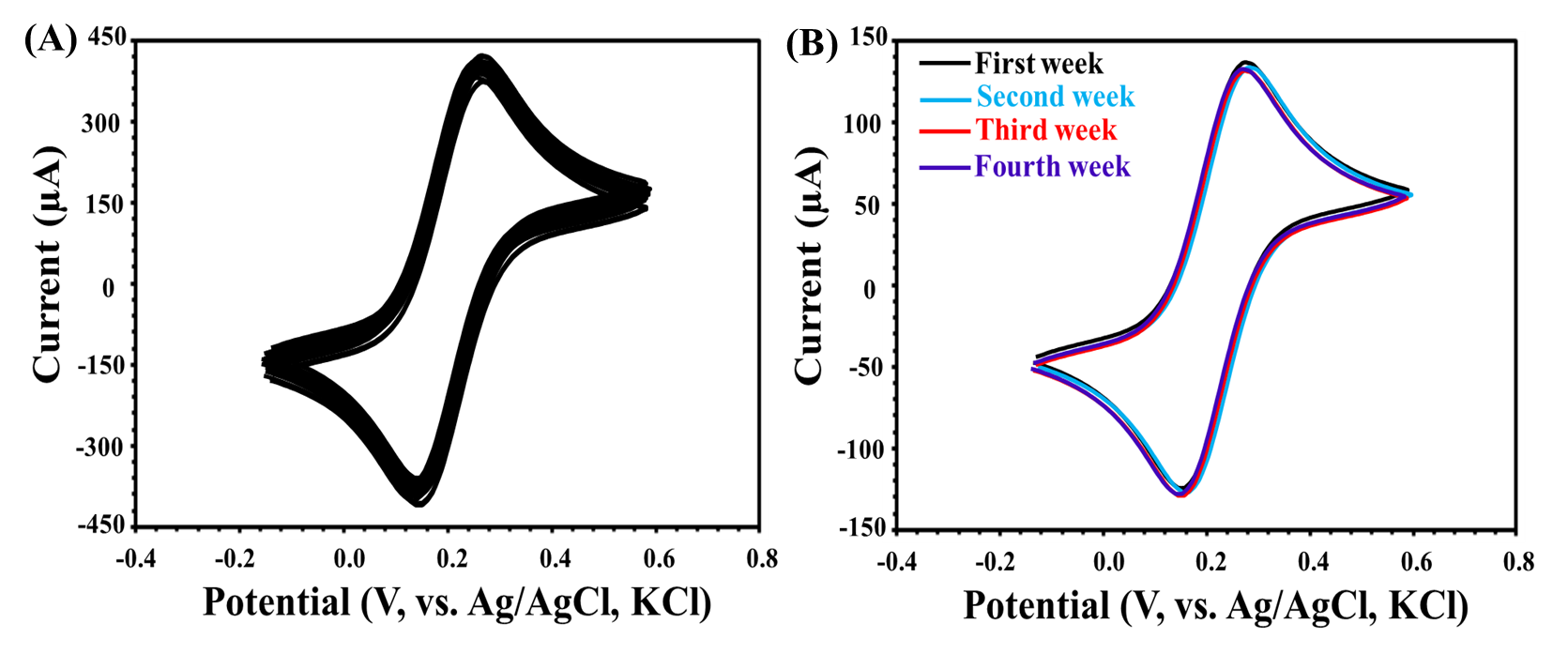


**Fig. S10** (A) CV scans of MIP-apt-AuNPs@ Fe_3_O_4_/GCE after impregnation in 0.1 M phosphate buffer containing 5.0 mM [Fe(CN)_6_]^3−/4−^ for 50 cycles at 300 mV s^-1^. (B) CV scans of MIP-apt-AuNPs@ Fe_3_O_4_/GCE after incubation with 10^3^ CFU mL^-^*^1^ S. aureus* at scan rate of 300 mV s^-1^.

**References**

1. H. Zhang, S. Yao, X. Song, K. Xu, J. Wang, J. Li, C. Zhao, M. Jin. One-step colorimetric detection of Staphylococcus aureus based on target-induced shielding against the peroxidase mimicking activity of aptamer-functionalized gold-coated iron oxide nanocomposites. Talanta 232 (2021) 122448.
